# Comprehensive Analysis of *Trypoxylus dichotomus*: Inorganic Elements, Amino Acids, and Bioactive Compounds and Their Anticancer, Antioxidant, and Neuroprotective Properties

**DOI:** 10.3390/molecules30020220

**Published:** 2025-01-08

**Authors:** Dongyan Ye, Qianhui Li, Xuwen Liu, Jie Zhou, Shuren Yin, Suyi Zhang, Jing Wang, Kh Ahammad Uz Zaman, Helong Bai, Fanlei Meng

**Affiliations:** 1 College of Chemistry, Changchun Normal University, Changchun 130032, China; yedongyan0214@163.com (D.Y.); qianyehns@163.com (Q.L.); jiajiayqsl@163.com (X.L.); zhoujie001119@163.com (J.Z.); 15560604768@163.com (S.Y.); yiyibuemo@163.com (S.Z.); zixi21@163.com (J.W.); 2 Technical Innovation Laboratory for Research and Development of Economic Plants and Edible and Medicinal Fungi in Cold Regions of Jilin Province, Changchun 130032, China; 3 DKI College of Pharmacy, University of Hawaii at Hilo, Hilo, HI 96720, USA; kzaman@hawaii.edu; 4 Institute of Agricultural Quality Standard and Testing Technology, Jilin Academy of Agricultural Sciences, Changchun 130033, China

**Keywords:** *T. dichotomus*, chemical components, anti-tumor, antioxidant, neuro-antioxidant

## Abstract

The Compendium of Materia Medica highlights the therapeutic properties of *Trypoxylus dichotomus* (*T. dichotomus*). In this study, the species and content of volatile components, inorganic elements, and amino acids were measured, and the activity of crude extracts of ethanol and water was studied. GC-MS analysis revealed 37–53 components across different life stages, excluding excessive heavy metals and containing essential trace elements. Amino acid profiling identified 15 types, rich in seven medicinal varieties. The in vitro anti-tumor testing of ethanol extracts from *T. dichotomus* adult males (TDA (M)) and *T. dichotomus* adult females (TDA (F)) was also performed. The experimental results showed that TDA (M) exhibited growth inhibition rates of 86.22% ± 1.19%, 85.42% ± 0.63%, 88.15% ± 3.27%, and 97.23% ± 0.60% against MKN-45, K-562, 239T, and 5637 tumor cells, respectively, while TDA (F) showed 81.28% ± 5.06% inhibition against K-562 cells. TDA best protected cells induced by H_2_O_2_ at the concentration of 12.5 μg/mL, achieving a cell survival rate of 81%. Overall, TDA, TDA(F), and TDA(M) have notable anti-tumor and antioxidant activity, highlighting the significance of chemical analysis for their potential bioactivity.

## 1. Introduction

*T. dichotomus* (Linnaeus, 1771) is a beetle belonging to the order Coleoptera, family Scarabaeidae, commonly known as the unicorn beetle [1,2]. The larval stage in certain insects is known as ‘chicken mother worms’. *T. dichotomus* is widely distributed in East Asia, including China, Japan, and Korea. It is not only kept as a popular pet for its ornamental value but is also highly valued for its medicinal properties. Both its larvae and adults are used in traditional medicine. The larvae and adults can suppress shock, alleviate blood stasis, relieve pain, mitigate poison, and provide laxative effects [3,4]. Studies have shown that *T. dichotomus* contains many active ingredients, such as triterpenoids, steroids, polysaccharides, fatty acids, vitamins, flavonoids, polyphenols, etc. These active ingredients have various types of pharmacological activity, including anti-inflammatory, antioxidant, immunomodulatory, anti-fatigue, and anti-tumor [3,5]. The medicinal value of *T. dichotomus* has attracted attention around the world [6].

In 1976, Japanese scholars extracted narone (dicotastin) from *T. dichotomus*; this compound had high inhibitory activity against solid tumor W-256 and marginal activity against P-388 lympholeukocytes [7]. The chemical composition of *T. dichotomus* has attracted the attention of many researchers. As an arthropod with inhibitory effects on tumor cells, *T. dichotomus* is often used in its entirety. Therefore, we hypothesized that there would be multiple bioactive substances.

In this paper, through the analysis of the chemical components, inorganic elements, total flavonoids, total polysaccharides, total polyphenols, total triterpenes, amino acid content, and antioxidant activity in model systems, as well as the nerve cell activity, we reveal the presence of numerous beneficial compounds. Further research and evaluation of other biological properties of *T. dichotomus* holds significant promise for future studies.

## 2. Results and Discussion

### 2.1. Analysis of GC-MS Results

According to the experimental results (Table 1), there were 44 components in the pupa, with 24 unique components; 37 components in the larva, with 16 unique components; 47 components in the female adult, with 18 unique components; and 53 components in the male adult, with 21 unique components. Comparing the volatile organic compounds (VOCs) at the different developmental stages of *T*. *dichotomus*, it was evident that the number of VOCs increased with the growth and morphological changes from larva to adult [8].

The adult stage has more VOCs than the larval stage due to its metamorphic development. Additionally, when both females and males are in the adult stage, only male adults have horns, resulting in more abundant VOCs in male adults than female adults. There are four VOCs shared among the pupa, larva, and adult stages: benzaldehyde, acetophenone, 2,5-bis-[(trimethylsilyl)oxy]-benzaldehyde, and 2-undecanone. Boldenone is a synthetic steroid hormone belonging to the steroidal family, exhibiting biological activity and pharmacological effects similar to those of natural hormones [9].

The eight common VOCs are organic compounds of aldehydes and ketones, both possessing aromatic properties, especially benzaldehyde and acetophenone. Ketones typically have a mild and fresh odor, while aldehydes usually have a strong, pungent smell. These substances all present distinctive odors, especially benzaldehyde, which has a strong almond or bitter almond aroma. Thus, the combined action of these four components may contribute to the distinct odor of *T*. *dichotomus*. Analyzing these four standard components can be highly useful in eliminating the distinctive odor of *T*. *dichotomus*.

### 2.2. Analysis of Experimental Results for Total Flavonoid Content 

Standard curves were obtained by using the absorbance as the ordinate and the rutin concentration as the abscissa, y = 1.4281x + 0.0454 (R^2^ = 0.9981). As can be seen from the data in Table 2, during the TDL (W) stage, the total flavonoid content was 155.7 mg/g, which may indicate a higher level of certain biochemical components or metabolites in *T. dichotomus* at this stage. In the TDP (W) stage, the total flavonoid content decreased significantly to 13.9 mg/g compared to the TDL (W) stage, possibly representing a reduction in certain biochemical processes. In the TDA (M, W) and TDA (F, W) stages, the total flavonoid content was 35.9 mg/g and 34.5 mg/g, respectively, within a similar range, indicating relatively stable biochemical characteristics during these two stages.

### 2.3. Analysis of Experimental Results for Total Polyphenol Content

Standard curves were drawn for the regression equations with the absorbance as the ordinate and the monohydrate gallic acid concentration as the abscissa, y = 4.6251x + 0.0235 (R^2^ = 0.9937). As can be seen from the data in Table 2, the total polyphenol content during the TDL (W) and TDA (M, W) stages was relatively close, at 25.8 mg/g and 25.5 mg/g, respectively. The total polyphenol content during the TDP (W) stage was slightly lower than that of the TDL (W) and TDA (M, W) stages, at 23.4 mg/g. The total polyphenol content during the TDA (F, W) stage was significantly higher than in the other stages, at 67.6 mg/g.

This difference may reflect significant variations or transformations in the total polyphenol content at different stages of *T. dichotomus* [10,11].

### 2.4. Analysis of Experimental Results for Total Triterpene Content

A standard curve was drawn with the absorbance as the ordinate and the oleanolic acid concentration as the abscissa to obtain the regression equation y = 36.415 x − 0.0338 (R^2^ = 0.9967). As can be seen from the data in Table 2, during the TDP (W) stage, the total triterpene content was the highest at 39.5 mg/g, significantly surpassing the other stages. The total triterpene content during the TDL (W) stage was 22.8 mg/g, at a moderate level. The total triterpene content during the TDA (F, W) and TDA (M, W) stages was 18.0 mg/g and 15.0 mg/g, respectively, being relatively lower.

This difference may reflect a decrease in the synthesis capacity of total triterpenes during the growth of *T. dichotomus* or insufficient compounds required for synthesis [12,13,14].

### 2.5. Analysis of Experimental Results for Total Polysaccharide Content 

Standard curves were obtained by taking the absorbance as the ordinate and the glucose concentration as the abscissa, y = 15.657x + 0.0906 (R^2^ = 0.9947). As can be seen from the data in Table 2, there were differences in the polysaccharide content among the different stages of *T. dichotomus*. The highest total polysaccharide content was observed during the larval stage, at 33.5 mg/g.

As *T. dichotomus* develops, there is a decrease in the polysaccharide content, which may reflect changes in the polysaccharide synthesis and metabolism capabilities during the growth and development of *T. dichotomus* [15,16].

### 2.6. Experimental Results from Amino Acid Content Analysis

As can be seen from the data in Table 3, both TDA (M) and TDA (F) were found to contain 14 types of amino acids, with the same species present but varying significantly in quantity. Among them, the levels of Asp, Thr, Ser, Glu, Cys, Met, Ile, Leu, Tyr, Phe, His, and Pro in TDA (M) were slightly higher than those in TDA (F), while the levels of Ala and Cys were slightly lower than those in TDA (F). The content of His was the highest in both TDA (M) and TDA (F), with content of 203.4 mg/g in TDA (M) and 217.17 mg/g in TDA (F). The Cys content was the lowest in both TDA (M) and TDA (F).

In most cases, there were differences in the content of various amino acids between TDA (M) and TDA (F), indicating potential differences in their metabolism and physiological characteristics. Asp, Glu, and Gly had relatively high content in both TDA (M) and TDA (F); these are common amino acids in proteins.

In TDA (M), the content of His was exceptionally high, reaching 217.17 mg/g, while, in TDA (F), the content of His slightly decreased to 203.48 mg/g but still remained high. This difference may be related to gender characteristics, growth and development, or other physiological processes. It is possible that, because TDA (M) and TDA (F) can synthesize His by themselves, there is high His content in the body.

The content of Tyr and Phe was relatively low in both TDA (M) and TDA (F). These two amino acids are aromatic amino acids, and their low content may be due to the preference of TDA (M) and TDA (F) to prioritize the synthesis or metabolism of other amino acids, resulting in relatively low levels of aromatic amino acids. The synthesis of these amino acids may be limited due to a lack of precursor substances or nutrients in the environment, resulting in lower content [12,17,18].

### 2.7. Analysis of Results of Inorganic Chemical Element Test

As can be seen from the data in Table 4, the analysis detected 23 elements in *T. dichotomus*, including essential trace metal elements for human health, such as Mn, Cu, Zn, Ni, and Mg. Among these, Be, Sn, Sb, and Ti were not detected in any of the stages (TDL, TDP, TDA (M), TDA (F)); Li and Pb were not detected in TDP and TDA (M); Al was not detected in TDP and TDA (F); B was not detected in TDL, TDA (M), and TDA (F); V and Cr were not detected in TDP, TDA (M), and TDA (F); and Co and Cd were not detected in TDA (M) and TDA (F).

According to the provided data, this dataset includes the levels of inorganic elements in TDL, TDP, TDA (F), and TDA (M).

Regarding Mg, it showed relatively high levels in the TDL, TDP, and TDA (F) stages, with lower levels in the TDA (M) stage; Ga, Al, Bi, Ti, Cr, Co, and Cu also exhibited relatively high levels in some stages of *T. dichotomus*; Li, V, Mn, Ni, As, Sr, and Cd were present in certain amounts in certain stages of *T. dichotomus*; Sn, Sb, Be, and Ti were either absent or present in very low amounts in different stages of *T. dichotomus*; and the content of Zn did not vary significantly across the different stages of *T. dichotomus*.

The analysis of the inorganic elements indicates that *T. dichotomus* does not contain heavy metal elements but contains essential inorganic elements required by the human body, thus posing no toxicity or harm to humans [19].

### 2.8. Analysis of Results of Anti-Tumor Activity Experiment

As can be seen from the data in Table 5, the results indicate that TDA (M, C) has inhibitory effects on MKN-45 (human gastric cancer cells), K-562 (human chronic myelogenous leukemia cells), 239T (human embryonic kidney cells), and 5637 (human bladder cancer cells) and the effects are significant, with the inhibition rates of MKN-45, K-562, 239T, and 5637 being 86.22% ± 1.19%, 85.42% ± 0.63%, 88.15% ± 3.27%, and 97.23% ± 0.60%, respectively. TDA (F, C) only has inhibitory effects on K-562, with a significant effect and an inhibition rate of 81.28% ± 5.06%.

For MKN-45, the inhibition rate of TDA (M, C) (86.22% ± 1.19%) is higher than that of Dox (79.97% ± 1.95%). This suggests that TDA (M, C) may be more effective than Dox in inhibiting MKN-45 cells. For K-562, the inhibition rate of TDA (M, C) (85.42% ± 0.63%) is slightly higher than that of TDA (F, C) (81.28% ± 1.19%), while Dox has the lowest inhibition rate (78.13% ± 0.52%). This indicates that TDA (M, C) and TDA (F, C) may have similar effects in inhibiting K-562 cells, while the effect of Dox is weaker. For 5637, the inhibition rate of TDA (M, C) (88.15% ± 3.27%) is lower than that of Dox (96.78% ± 0.37%). This suggests that TDA (M, C) may have a weak inhibitory effect on 5637 cells. For 239T, the inhibition rate of TDA (M, C) (97.23% ± 0.60%) is slightly higher than that of Dox (95.84% ± 1.32%). This means that TDA (M, C) and Dox may have similar effects in inhibiting 239T cells.

TDA (M, C) exhibits relatively good inhibitory effects on different tumor cell lines, but the choice of a specific drug needs to consider other factors, such as the drug’s side effects and resistance differences in individual patients. In summary, the activity of *T*. *dichotomus* is significant, and it has a good inhibitory effect on the proliferation of the above-mentioned tumor cells.

### 2.9. Analysis of Experimental Results on In Vitro Antioxidant Activity 

**Figure 1:**
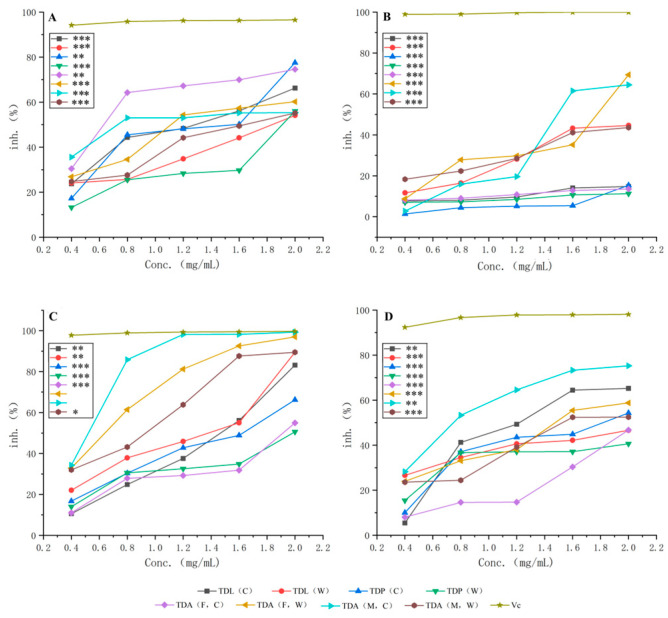
Concentration-dependent free radical scavenging activity of *T. dichotomus* extracts against DPPH (**A**), OH (**B**), ABTS^+^ (**C**), and O_2_^−^ (**D**) (compared with Vc: *** *p* < 0.001, ** *p* < 0.01, * *p* < 0.05). (The number of experiments was 3).

The antioxidant activity of the ethanol and water extracts from *T. dichotomus* was determined using four types of free radical scavenging assays, with Vc serving as a positive control. All exhibit certain antioxidant abilities, capable of scavenging free radicals (Figure 1). All samples of *T. dichotomus* show linear increases in their ability to scavenge four types of free radicals with increasing sample concentrations, with the overall trends being lower than that of Vc [11]. However, when the sample concentration is 1.2 mg/mL, the ABTS^+^· scavenging activity of TDA (M, C) is comparable to that of Vc, especially at 2 mg/mL, and the ABTS^+^· scavenging activity of TDA (F, C) is also comparable to that of Vc. At different concentrations, they show differences in their ability to scavenge ABTS^+^·, ·O_2_^−^, DPPH·, and ·OH. At a concentration of 2 mg/mL, these samples exhibit the highest ABTS^+^· scavenging activity, but the ·O_2_^−^ scavenging activity shows a decreasing trend at high concentrations. Although the free radical scavenging capacity of the samples of *T. dichotomus* is not as great as that of Vc, they demonstrate good antioxidant potential. Overall, while the antioxidant capacity of Vc is significantly stronger than that of the samples of *T. dichotomus*, some samples perform comparably to Vc in terms of their antioxidant capacity at specific concentrations [20]. According to the data shown in Figure 1A, the *p* values of TDP (C) and TDA (F, C) in DPPH· were less than 0.01, while the others were less than 0.001. In ·OH, all values were less than 0.001 (Figure 1B). In the ·O_2_^−^ radical scavenging experiment (Figure 1D), the *p* values of TDP (C) and TDA (M, C) were less than 0.01, and the others were less than 0.001. These results indicate that the scavenging activity of all extracts regarding DPPH·, ·OH, and·O_2_^-^ was significantly lower than that of Vc. In the ABTS^+^· experiment (Figure 1C), the *p* values of TDA (F, C) and TDA (F, W) were greater than 0.05, the *p* value of TDA (M, W) was less than 0.05, the *p* value of TDL (W) was less than 0.01, and the others were less than 0.001. This means that the scavenging activity of TDA (F, C) and TDA (F, W) for ABTS^+^·, reaching 2.0 mg/mL, was similar to that of Vc. Meanwhile, TDA (M, W) differed significantly compared to Vc, and the other differences were very significant when compared to Vc.

In Table 6, we present the IC_50_ values of the four radicals, where TDA (F, C) has the smallest IC_50_ value for DPPH· scavenging activity, and the value is 0.672 ± 0.306 mg/mL. For only TDA (F, W) and TDA (M, C), the IC_50_ value could be calculated for the ·OH scavenging activity, and the size of the difference was small. TDA (M, C) had the minimum IC_50_ value for both ABTS^+^· and ·O_2_^−^ scavenging activity, and the values were 0.479 ± 0.047 mg/mL and 0.773 ± 0.133 mg/mL, respectively. Compared to Vc, these values are relatively large.

### 2.10. Analysis of Neuro-Antioxidant Activity Detection Results

**Figure 2:**
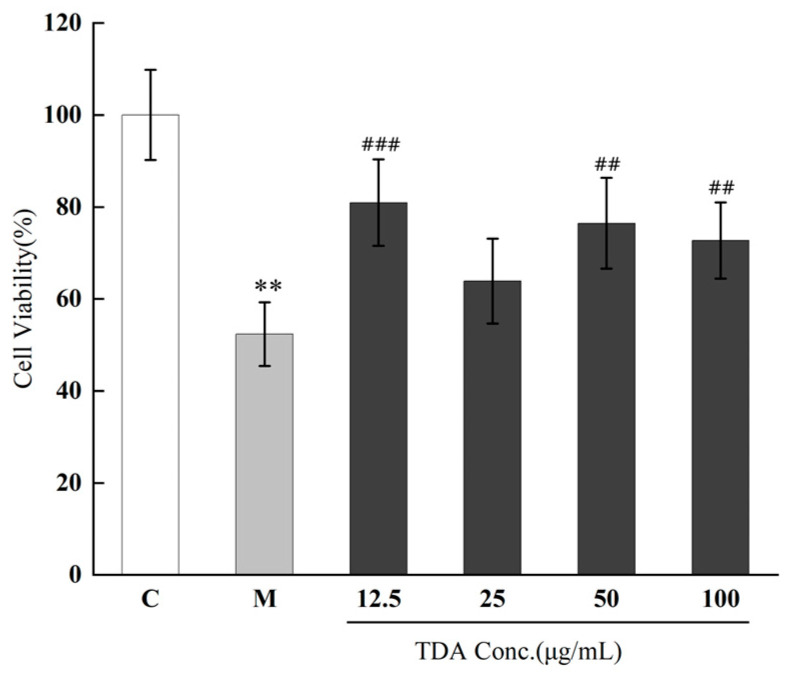
Effects of different concentrations of TDA on cell survival of oxidatively damaged PC12 cells (compared with control group: ** *p* < 0.01, compared with model group: ^###^ *p* < 0.001, ^##^ *p* < 0.01, data are means ± SD, n = 6). (Note: C represents the control group, M represents the model group, and TDA refers to the ethanol extract of the adult *T. dichotomus*).

As shown in Figure 2, TDA ethanol exhibits a certain protective effect on cells against H_2_O_2_-induced damage. At a concentration of 12.5 μg/mL of *T. dichotomus*, the protective effect on cells is the strongest, with a cell survival rate of up to 81%. The cell survival rate in the control group is 100%. After adding different concentrations of TDA, the degree of oxidative damage is alleviated, with cell survival rates of 81%, 64%, 77%, and 73%, respectively, indicating that TDA possesses a certain neuro-antioxidant effect. As the concentration of TDA increases, the degree of oxidative damage shows a decreasing trend, indicating that the antioxidant effect of TDA is concentration-dependent. Under experimental conditions, TDA ethanol exhibits a certain protective effect against hydrogen peroxide-induced oxidative stress, helping to alleviate the oxidative damage to neuronal cells. This neuro-antioxidant activity aids in protecting neuronal cells from oxidative damage and has potential applications in neuroprotection and the treatment of neurodegenerative diseases [21].

## 3. Experimental Section

### 3.1. Chemical and Reagents

The drugs used in this experiment were all of analytical purity. DPPH (1,1-diphenyl-2-picrylhydrazyl), ABTS (2,2′-azino-bis (3-ethylbenzothiazoline-6-sulfonic acid)), NBT (nitro blue tetrazolium chloride), NADH (nicotinamide adenine dinucleotide, reduced disodium salt), PMS (phenazine methosulfate), vanillin, and rutin were purchased from Shanghai Yuan Ye Co., Ltd (Shanghai, China). Potassium persulfate, salicylic acid, ferrous sulfate, and ascorbic acid were purchased from Shanghai Yi En Chemical Technology Co., Ltd (Shanghai, China). *T. dichotomus* was collected by Changchun Guoxin Modern Agricultural Science and Technology Development Co., Ltd (Changchun, China). and identified by Professor Liu Zhiwen as *T. dichotomus*.

### 3.2. Sample Grinding

The dried TDL, TDP, TDA(M), and TDA(F) were ground using a pulverizer and passed through a 40-mesh sieve to obtain the powder form; placed in a sealed bag; stored at 2–8 °C away from light; and set aside.

### 3.3. Extraction Procedure

One gram of a powdered sample was mixed with 50 mL distilled water and subjected to ultrasonication (600 W, 30 °C, 30 min) 3 times. The combined filtrate was then concentrated to 50 mL by rotary evaporation, followed by adding 200 mL anhydrous ethanol for 24 h to precipitate. The precipitate was centrifuged, dried, and obtained as the aqueous extract. The residue from the above filtration was mixed with 50 mL 70% ethanol and subjected to ultrasonication (600 W, 30 °C, 30 min) 3 times. The combined filtrate was concentrated by rotary evaporation and dried to obtain the ethanol extract.

TDL denotes the larvae of *T. dichotomus*, TDP denotes the pupae of *T. dichotomus*, TDA(F) denotes female adults of *T. dichotomus*, TDA(M) denotes male adults of *T. dichotomus*, TDL(W) and TDL(C) denote the water extract and ethanol extract of *T. dichotomus* larvae, TDP(W) and TDP(C) denote the water extract and ethanol extract of *T. dichotomus* pupae, TDA(F, W) and TDA(F, C) denote the water extract and ethanol extract of female *T. dichotomus* adults, and TDA(M, W) and TDA(M, C) denote the water extract and ethanol extract of male *T. dichotomus* adults.

### 3.4. GC-MS Analysis

A gas chromatography–mass spectrometry system (7890A-5975C) (Jinko Ruida Technology Co., Ltd., Beijing, China) equipped with a quartz capillary column (HP-5MS, 30 m × 25 mm ID × 0.25 mm) (Haolian Technology Development Co., Ltd., Tianjin, China) was used to analyze two replicates of each sample. Here, 5 g of sample powder (see Section 2.3) was placed into a 20 mL solid-phase microextraction sampling bottle and sampled at 110 °C in an oven for 30 min. It was then inserted into an injector with an extraction head at 60 °C for 30 min. After extraction, it was immediately inserted into the inlet and tested.

GC conditions: inlet temperature—250 °C, no split; carrier gas flow rate—1.0 mL/min; oven temperature—initial temperature 40 °C, maintained for 15 min, ramped at 4 °C/min to 180 °C, maintained for 2 min, then ramped at 50 °C/min to 230 °C, maintained for 4 min, and finally ramped at 50 °C/min to 250 °C.

MS conditions: electron impact (EI) source, 70 eV, full scan (SCAN) and selected ion monitoring (SIM); scan range (*m*/*z*)—50–500; ion source temperature—230 °C.

### 3.5. Total Flavonoid Content Determination

The total flavonoid content in the extract of *T. dichotomus* was measured by the sodium nitrite–aluminum nitrate method [22]. Here, 0.2 mg/mL standard solution of rutin was prepared with 70% ethanol and diluted into different concentrations (0, 0.1, 0.12, 0.14, 0.16, 0.18, 0.20 mg/mL) according to a gradient. Then, 0.3 mL of 5% NaNO_2_ was added, and the sample was mixed well and allowed to stand for 6 min. Next, 0.3 mL of 5% Al(NO_3_)_3_ was added, and the sample was mixed well and allowed to stand for another 6 min. Subsequently, 2.0 mL of 4% NaOH was added, followed by the addition of 1.4 mL of 70% ethanol; the sample was mixed well and allowed to stand for 15 min. The absorbance was measured at 510 nm. The extract of *T. dichotomus* was processed using the above method, and the absorbance of the sample solution was measured at 510 nm to calculate the total flavonoid content in the extract of *T. dichotomus.*

### 3.6. Total Polyphenol Content Determination

The Folin–Ciocalteu colorimetric method was used to determine the polyphenol content [12]. A certain amount of gallic acid was weighed and distilled water was added to achieved a concentration of 0.08 mg/mL; this was diluted to different concentrations (0, 0.016, 0.032, 0.048, 0.064, 0.08 mg/mL). Then, 0.5 mL of 10% Folin–phenol reagent and 1 mL of 10% Na_2_CO_3_ solution were added. To ensure accuracy, three replicates were set for each gradient. After thorough mixing, the mixtures were allowed to react in the dark for 30 min, and the absorbance at 760 nm was measured. The extract of *T. dichotomus* was processed according to the above method, and the absorbance of the sample solution was measured at 760 nm to calculate the total polyphenol content in the extract of *T. dichotomus*.

### 3.7. Total Triterpene Content Determination

The total triterpene content was determined using the vanillin–perchloric acid colorimetric method [9]. Oleanolic acid was weighed and dissolved in methanol with a concentration of 0.15 mg/mL and then diluted to different concentrations (0, 0.03, 0.06, 0.09, 0.12, 0.15 mg/mL). The tubes were then evaporated to dryness in a water bath, followed by the addition of 0.023 mL of freshly prepared 5% vanillin–glacial acetic acid solution and 0.075 mL of HClO_4_. The tubes were then placed in a 75 °C water bath for 15 min, cooled, and diluted to 0.25 mL with glacial acetic acid. After thorough mixing, the absorbance of the sample solution was measured at 550 nm. The extract of *T. dichotomus* was processed using the above method, and the absorbance of the sample solution was measured at 550 nm, allowing the total triterpene content in the extract of *T. dichotomus* to be calculated.

### 3.8. Total Polysaccharide Content Determination

The total polysaccharide content in the extract of *T. dichotomus* was determined using the phenol–sulfuric acid method [15]. Glucose with a concentration of 0.04 mg/mL was prepared and diluted to different concentrations (0, 0.008, 0.016, 0.024, 0.032, 0.04 mg/mL) according to the gradient. Then, 0.2 mL of 6% phenol solution was added to each tube, followed by the slow addition of 1 mL of concentrated sulfuric acid. After thorough mixing, the tubes were allowed to react in the dark for 20 min. Upon completion of the reaction, the absorbance was measured at a wavelength of 490 nm using a UV–Vis spectrophotometer. The extract of *T. dichotomus* was processed using the above method, and the absorbance of the sample solution was measured at 490 nm, allowing the total polysaccharide content in the extract of *T. dichotomus* to be calculated.

### 3.9. Amino Acid Content Determination

An amino acid analyzer (S433D, Scham GmbH) was used to analyze two replicates of each sample [17]. First, 50 mg of TDA (M) and TDA (F) powder (see Section 2.2) was separately weighed into 20 mL anaerobic hydrolysis tubes. Then, 10 mL of 6 mol/L HCl was added and the tubes were filled with high-purity nitrogen for 30 s. The caps were tightly closed and the sealed hydrolysis tubes were placed in a constant-temperature drying oven at 110 °C for 22 h. After cooling, the contents were mixed and the tubes were opened. The mixture was filtered using filter paper and 0.5 mL of the filtrate was placed in a water bath with a nitrogen blower. The temperature was set to 50 °C and nitrogen was then blown until dry. Two to three drops of ultrapure water were then added and the drying process was repeated once. After adding 3 mL of sample diluent, the solution was filtered through a 0.22 µm membrane filter. The amino acid types and content in the extract of *T. dichotomus* were determined using an automatic amino acid analyzer. The detector utilized was a photodiode array (VIS), with wavelengths of 570 and 440 nm; at 440 nm, 14 amino acids could be detected, namely Asp, Thr, Ser, Glu, Cys, Met, Ile, Leu, Tyr, Phe, His, Asp, and Gly, and only one could be detected at 570 nm. The chromatographic column (4.6 × 150 mm, 7 μm) was a cation exchange column with a column temperature ranging from 40 °C to 70 °C; the injection volume was 50 μL; and the post-column reaction chamber temperature was 115 °C.

### 3.10. Inorganic Element Determination

ICP-MS (ICAPTQ, Thermo Fisher Scientific Inc., Waltham, MA, USA) was used to analyze two replicates of each sample [8]. The sample pretreatment involved accurately weighing 50 mg of the sample powder (see Section 2.2) and placing it into a digestion flask, to which 2 mL of nitric acid was added. A blank control group without samples was also prepared. The flask was placed into a microwave digestion instrument for digestion for 1.5 h. After digestion, when the flask had cooled to room temperature, it was transferred to an acid-chasing instrument and chased with acid until the volume of nitric acid was approximately 1 mL; this indicated the completion of the acid chasing. The liquid from the digestion flask was transferred to a 50 mL volumetric flask, diluted with ultrapure water, and filtered through a 0.45 μm membrane filter for subsequent detection. Due to the high content of certain elements, the samples were gradient-diluted with ultrapure water, with 50-fold and 10-fold dilutions conducted before testing.

The ICP-MS instrument was then adjusted and optimized to ensure that the conditions were optimal. The operating parameters of the instrument were set as follows: plasma radiofrequency power at 1550 W, carrier gas flow rate at 0.8 L/min, nebulizer flow rate at 1.5 L/min, auxiliary gas flow rate at 1.5 L/min, cooling gas flow rate at 20 L/min, and analysis time at 0.6 s.

Standard samples: A multi-element solution (GBW(E)081535) was prepared by adding 0.02 mL of the standard stock solution to 3.98 mL of ultrapure water, resulting in a mixed standard solution with a concentration of 500 ng/mL. This solution was further diluted to prepare mixed standard solutions with concentrations of 0, 1, 2, 5, 10, and 20 ng/mL, which were used to construct the standard curve.

Internal standard solution: 0.02 mL of the internal standard stock solution was taken and diluted to 100 mL to prepare the internal standard solution.

### 3.11. In Vitro Anti-Tumor Activity Assay

The cell counting kit (CCK-8) assay was used to evaluate the proliferation inhibition activity of the compounds on cells [23].

Cell culture: The CSC-20 cells were cultured in complete medium containing 10% fetal bovine serum and a penicillin–streptomycin sulfate double-antibody mixture (100×) (PB180120), and the cells were combined into a single-cell suspension. In each well of a 96-well plate, 90 μL of cells at a concentration of 5 × 10^4^ cells/mL for adherent cells and 9 × 10^4^ cells/mL for suspension cells were seeded and pre-incubated for 24 h at 37 °C in a 5% CO_2_ environment. Each well was then treated with 10 μL of the test compound solution.

For the initial screening of the activity, each sample was tested at one concentration with three replicate wells and incubated in a culture chamber for 48 h. The experiment included blank, control, and drug groups. The old culture medium and drug solution were removed from the adherent cells (the CCK-8 solution was directly added to the suspension cells) and then each well was treated with 100 μL of CCK-8 solution diluted tenfold. The cells were further incubated at 37 °C in a 5% CO_2_ environment for 1–4 h (light-avoiding operation, real-time observation). The absorbance at 450 nm was measured using an enzyme-linked immunosorbent assay (ELISA) reader, and the cell proliferation inhibition rate was calculated according to the following formula:

$$Inhibition\;rate(\%)=\frac{OD_{control}-OD_{drug}}{OD_{control}-OD_{blank}}\times 100\%$$

where *OD_control_* is the optical density of the control group, *OD_drug_* is the optical density of the drug group, and *OD_blank_* is the optical density of the blank group. The positive control used was doxorubicin hydrochloride (Dox).

### 3.12. DPPH Scavenging Rate Assay

The scavenging abilities for DPPH radicals were assessed by UV absorbance [24]. Here, 0.1 mL of different concentrations of *T. dichotomus* solution was added to 0.1 mL of 75% ethanol solution of DPPH, mixed well, and left to react in the dark for 30 min. The absorbance was then measured at 517 nm. The control group used 75% ethanol instead of DPPH 75% ethanol solution; the blank group used 0.1 mL of distilled water instead of the sample; and ascorbic acid (Vc) was used as a positive control. The formula for the calculation of the DPPH· scavenging rate was as follows:

$$I=\frac{1-(A_{2}-A_{1})}{A_{0}}\times 100\%$$

In this formula used to represent the scavenging rate, *A*_2_ denotes the sample solution containing the blank solvent, *A*_1_ denotes the sample solution containing the working solution of free radicals, and *A*_0_ denotes the blank solvent containing the working solution of free radicals.

### 3.13. ·OH Scavenging Rate Assay

The scavenging abilities for OH radicals were assessed by UV absorbance [25]. To 0.1 mL of different concentrations of *T. dichotomus* solution, we added 0.05 mL of 9 mmol/L FeSO_4_·7H_2_O, 0.05 mL of salicylic acid, and 0.05 mL of H_2_O_2_ solution. After incubation at 37 °C for 30 min in a water bath, the sample was left to stand for 30 min and the absorbance was measured at 510 nm. For the control group, distilled water was used instead of the H_2_O_2_ solution; the blank group used distilled water instead of the sample solution, and Vc was used as a positive control. The formula for the calculation of the ·OH scavenging rate was as follows:

$$I=\frac{1-(A_{2}-A_{1})}{A_{0}}\times 100\%$$

Again, in this formula representing the scavenging rate, *A*_2_ refers to the sample solution containing the blank solvent, *A*_1_ refers to the sample solution containing the working solution of free radicals, and *A*_0_ refers to the blank solvent containing the working solution of free radicals.

### 3.14. ABTS^+^·Scavenging Rate Assay

The scavenging abilities for ABTS^+^ radicals were assessed by UV absorbance [17]. Here, 0.05 mL of different concentrations of *T. dichotomus* solution was added to 0.2 mL of ABTS^+^ solution and reacted under light avoidance for 6 min, with the absorbance measured at 734 nm. The control group used distilled water instead of the ABTS^+^ solution; the blank group used distilled water instead of the sample solution; and Vc was used as a positive control. The formula for the calculation of the ABTS^+^· scavenging rate was as follows:

$$I=\frac{1-(A_{2}-A_{1})}{A_{0}}\times 100\%$$

In this formula representing the scavenging rate, *A*_2_ is again the sample solution containing the blank solvent, *A*_1_ is the sample solution containing the working solution of free radicals, and *A*_0_ is the blank solvent containing the working solution of free radicals.

### 3.15. ·O_2_^−^ Scavenging Rate Assay

The scavenging abilities for O_2_^-^ radicals were assessed by UV absorbance [10]. First, 0.05 mL of different concentrations of the solution of *T. dichotomus* was then added to 0.05 mL of 0.3 mmol/L NBT, 0.05 mL of 0.9 mmol/L NADH, and 0.05 mL of 0.2 mmol/L PMS solutions, respectively. These were reacted in a 37 °C water bath for 5 min and the absorbance was measured at 510 nm. The control group used distilled water instead of the NBT solution; the blank group used distilled water instead of the sample solution; and Vc was used as a positive control. The formula for the calculation of the ·O_2_^−^ scavenging rate was as follows:

$$I=\frac{1-(A_{2}-A_{1})}{A_{0}}\times 100\%.$$

In this formula representing the scavenging rate, *A*_2_ is the sample solution containing the blank solvent, *A*_1_ is the sample solution containing the working solution of free radicals, and *A*_0_ is the blank solvent containing the working solution of free radicals.

### 3.16. Neuro-Antioxidant Activity Assay

An experiment using PC12 neuronal cells was performed. PC12 cells were cultured in complete medium containing 10% fetal bovine serum, 100 μg/mL penicillin, and 100 μg/mL streptomycin and placed in a 37 °C, 5% CO_2_ humidified incubator. After 2–3 days, the cells were passaged: the original medium was discarded and the cells were washed once with 0.5 mL PBS to remove the fetal bovine serum. Then, 0.5 mL trypsin was added, and, after 2 min of digestion in a laminar flow hood, 1 mL complete medium was added to stop the digestion. The cells were then evenly distributed into new culture vessels for further cultivation.

The MTT assay was used to evaluate the protective effects of different concentrations of *T. dichotomus* against H_2_O_2_-induced cell damage [13]. PC12 cells were seeded at a density of 1 × 10^5^ cells/mL, 100 μL per well, in a sterile 96-well plate and cultured in the incubator for 24 h. After observing the cell adhesion under a microscope, the culture medium in each well was discarded. The experiment consisted of a control group, a model group, and a *T. dichotomus* treatment group, with 6 replicate wells per group. The control group contained only the culture medium, the model group received an equal amount of 600 μmol/L H_2_O_2_, and the *T. dichotomus* treatment group contained concentrations of 12.5, 25, 50, and 100 μg/mL of *T. dichotomus*, along with 600 μmol/L H_2_O_2_. After 24 h of incubation, the culture medium was removed and 10 μL of MTT working solution was added to each well. The cells were further incubated for 3–4 h; then, the old medium was aspirated and 100 μL of DMSO was added to each well. After shaking the plate for 10 min on a microplate shaker, the absorbance (A) at 490 nm was measured using an enzyme-linked immunosorbent assay reader. Cell viability was calculated as follows: $\mathrm{Cell}\;\mathrm{vivability}\;(\%)=\frac{A_{\mathrm{drug}}}{A_{\mathrm{control}}}\times 100\%$.

## 4. Conclusions

The analysis of the chemical composition of *T. dichotomus* reveals essential findings. The detection of inorganic elements indicates that *T. dichotomus* does not contain excessive heavy metal elements but does contain essential inorganic elements that are necessary for human health. The determination of the total polyphenols, total polysaccharides, total flavonoids, and total triterpenoids at different developmental stages in this turtle show that the content of these compounds is influenced by the developmental stage, with significant differences observed among other compounds. The analysis of the amino acids reveals that *T. dichotomus* is rich in medicinal amino acids, with high levels of histidine, glutamic acid, glycine, and aspartic acid, making it a suitable source for the extraction of these amino acids. The extract of *T. dichotomus* exhibits notable antioxidant, anti-tumor, and neuro-antioxidant activity. Additionally, the *T. dichotomus* samples demonstrated specific abilities to scavenge free radicals, albeit generally lower than those of Vc. However, at specific concentrations, some *T. dichotomus* samples exhibited free radical scavenging abilities comparable to Vc, indicating significant antioxidant potential. Moreover, the TDA ethanol extract showed good anti-tumor activity against A-673. The TDA (M) ethanol extract showed good anti-tumor activity against MKN-45, K-526, 5637, and 239T. The TDA (F) ethanol extract showed high anti-tumor activity against K-562. More research on TDA, TDA (M), and TDA (F)’s grading is needed to identify and isolate additional biological properties for the evaluation of their in vitro and in vivo biological activity. More research is also required on the ethanol extract and water acetate fraction to identify and isolate additional bioactive components in order to assess their in vitro and in vivo biological activity.

## Figures and Tables

**Table 1:** The chemical constituents of different *T*. *dichotomus* powders.

No.	Name	RT	Percentage			
			TDL	TDP	TDA (F)	TDA (M)
1	Dimethyl disulfide	4.176	—	—	7.773	6.515
2	6,7-Dioxabicyclo[3.2.2]non-8-ene	4.199	—	1.612	—	—
3	2,5-Dimethyl-pyrazine	14.087	—	—	6.084	—
4	2-Methyl-butanoic acid	16.192	—	—	1.188	—
5	N-(2-Methylbutylidene)isobutylami	17.348	—	—	1.518	—
6	Benzaldehyde	18.756	4.531	3.59	12.301	12.271
7	Dimethyl trisulfide	19.025	—	1.597	19.965	21.11
8	Benzonitrile	20.598	—	—	1.26	—
9	4,5-Dihydro-3-phenyl-6H-1,2,5-oxadiazine-6-thione	20.661	—	—	—	0.879
10	1-Octen-3-ol	20.81	5.895	11.732	—	—
11	3-Octanone	21.136	4.953	10.97	—	—
12	(2-Endo,5-exo)-1,7,7-trimethyl-bicyclo[2.2.1]heptane-2,5-diol	21.308	—	—	—	1.525
13	3-Octanol	21.897	11.742	31.765	—	—
14	2-Ethyl-6-methyl-pyrazine	21.949	—	—	0.49	0.978
15	1,4-Dichloro-benzene	22.223	—	—	2.423	4.517
16	2-Acetylthiazole	23.03	1.813	—	—	—
17	2-Methyl-N-(2-methylbutylidene)-1-butanamine	24.392	—	—	1.254	1.979
18	Benzeneacetaldehyde	24.541	1.401	8.546	—	—
19	3-Methyl-N-(3-methylbutylidene)-1-butanamine	24.855	—	—	—	1.714
20	3,5-Bis(morpholinomethyl)-4-oxo-2,2,6,6-tetramethylpiperidine-1-oxyl	25.222	0.924	—	—	—
21	Acetophenone	25.737	1.309	1.767	1.475	1.972
22	2-Ethyl-3,5-dimethyl-pyrazine	26.561	—	—	0.368	0.586
23	Tetramethyl-pyrazine	26.852	—	—	3.493	4.387
24	4-Methyl-phenol	27.018	4.064	3.484	—	—
25	2-Nonanone	27.207	—	—	0.83	1.141
26	Nonanal	27.808	4.341	2.796	—	—
27	Phenylethyl alcohol	28.277	—	1.971	—	—
28	2,5-Bis[(trimethylsilyl)oxy]-benzaldehyde	28.867	0.877	0.643	0.717	0.897
29	Benzyl nitrile	29.381	—	—	3.722	3.037
30	N-(3-Methylbutyl)acetamide	29.759	—	—	0.624	0.562
31	2-Methylisoborneol	31.115	0.796	—	—	—
32	Tricyclo[4.3.1.1(3,8)]undecan-1-ol	31.327	—	—	0.902	0.825
33	Nonanenitrile	31.43	—	—	0.97	—
34	2-Hydroxy-4-methyl-benzaldehyde	31.779	—	—	1.335	1.317
35	2-Decanone	31.939	1.338	—	1.703	2.277
36	Dodecane	32.174	—	—	0.441	—
37	6-Heptyltetrahydro-2H-pyran-2-one	32.202	—	—	0.395	—
38	Pterin-6-carboxylic acid	32.42	—	—	—	0.609
39	Decanal	32.563	1.699	1.536	—	—
40	Dimethyl-tetrasulfide	32.717	—	—	0.72	
41	N-[5-Hydroxy-n-pentyl]-arachidonic amide	32.74	—	—	—	0.559
42	5-Propyl-1,3-benzodioxole	32.769	1.895	—	—	—
43	2-Azido-2,4,4,6,6-pentamethylheptane	32.906	—	—	0.528	—
44	Hexylresorcinol	33.118	—	0.255	—	—
45	Geranyl isovalerate	33.301	—	—	0.642	—
46	3-Methoxy-2,4,6-trimethyl-cyclohex-2-enone	33.61	—	0.603	—	—
47	Benzenepropanol	33.982	1.404	0.437	—	—
48	5,7-Dodecadiyn-1,12-diol	34.182	—	0.358	—	—
49	Pyrrolizin-1,7-dione-6-carboxylic acid methyl ester	34.285	—	—	0.438	—
50	2-Myristynoyl pantetheine	34.394	—	—	0.355	—
51	3-Ethyl-5-(2-ethylbutyl)-octadecane	35.138	—	—	0.45	0.32
52	(Z)-3-Decen-1-ol	35.252	—	0.432	—	—
53	(2-Isopropyl-phenoxy)silyloxy-silane	35.452	—	—	2.304	0.428
54	2-Phenyl-l-p-toluenesulfonylaziridine	35.464	—	—	—	1.755
55	3,3-Dimethyl-5-oxo-cyclohexanecarboxaldehyde	35.67	—	0.406	—	—
56	(E)-10-Heptadecen-8-ynoic acid methyl ester	35.71	—	—	—	0.051
57	2-Trimethylsiloxy-6-hexadecenoic acid methyl ester	35.825	0.833	—	—	—
58	(E)-3-Decen-1-ol	35.876	—	1.773	—	—
59	3-Trifluoroacetoxytetradecane	35.99	—	1.023	—	—
60	(Z)-7-Hexadecenal	36.202	—	0.223	—	—
61	5-Acetyl-4,6,6-trimethylcyclohexa-2,4-dienone	36.305	—	—	—	0.085
62	Hexahydro-4,4,7a-trimethyl-2(3H)-benzofuranone	36.54	—	0.105	—	—
63	(7R,8R)-Ethyl-8-hydroxy-trans-bicyclo[4.3.0]-3-nonene-7-carboxylate	36.557	—	—	1.081	—
64	1,2,3,4-Tetrahydro-5-methyl-naphthalene	36.597	0.719	—	—	—
65	4-Methyl-3-heptanone	36.74	—	0.153	—	—
66	2-Undecanone	36.94	0.781	0.298	1.344	1.287
67	Tridecane	37.146	—	1.128	0.82	1.321
68	3-(2,5-Dimethylanilinomethyl)-5-(3-fluorobenzylidene)-2,4-thiazolidinedione	37.221	—	—	0.644	—
69	tert-Hexadecanethiol	37.524	—	—	0.829	—
70	Trichloroacetic acid-hexadecyl ester	37.547	—	—	—	0.784
71	Undecanal	37.558	1.386	—	—	—
72	4-Hydroxy-4-methylhex-5-enoic acid-tert-butyl ester	37.575	—	1.22	—	—
73	4-(3-Hydroxy-2,6,6-trimethylcyclohex-1-enyl)pent-3-en-2-one	37.804	—	—	0.492	—
74	10-Methyl-tricyclo[4.3.1.1(2,5)]undec-3-en-10-ol	37.827	—	—	—	0.227
75	1,2,3,4-Tetrahydro-2,7-dimethyl-naphthalene	38.949	0.787	—	—	—
76	Boldenone	38.983	—	—	—	0.618
77	2,2′-Diethyl-1,1′-biphenyl	39.572	0.694	—	—	—
78	3,7,11-Trimethyl-1-dodecanol	39.658	—	—	—	0.389
79	Geranyl isovalerate	39.887	—	—	—	0.13
80	4,4,6-Trimethyl-6-phenyltetrahydro-1,3-oxazine-2-thione	40.425	—	—	—	0.149
81	Phytol	40.556	—	0.299	—	—
82	3-Methyl-N-(2-phenylethylidene)-1-butanamine	40.957	—	—	7.205	4.813
83	1,2,3,4-Tetrahydro-6,7-dimethyl-naphthalene	41.266	1.714	—	—	—
84	Tetradecane	41.735	—	—	1.012	1.482
85	trans-1,10-Dimethyl-trans-9-decalinol	41.753	8.485	—	—	—
86	(4α,4α,8β)-Octahydro-4,8-dimethyl-4(2H)-naphthalenol	41.769	—	1.307	—	—
87	trans-5-(Hexadecyloxy)-2-pentadecyl-1,3-dioxane	41.872	—	—	0.582	0.317
88	Dodecanal	42.21	—	0.324	—	—
89	4-(Hexadecyloxy)-2-pentadecyl-1,3-dioxane	42.302	—	—	1.308	1.142
90	5α-Cholestan-2-one oxime	42.868	—	—	—	0.106
91	1-(5-Ethyl-tetrahydrofuran-2-yl)-3,3-dimethyl-butan-2-one	43.389	—	0.113	—	—
92	1,2,3,4-Tetrahydro-2,5,8-trimethyl-naphthalene	43.481	1.783	—	—	—
93	(E)-6,10-Dimethyl-5,9-undecadien-2-one	44.07	—	—	1.678	1.593
94	2,6,10-Trimethyl-tetradecane	44.407	0.947	—	0.46	0.819
95	1-(1,6-Dioxooctadecyl)-pyrrolidine	44.928	—	—	2.001	1.549
96	1,12-Tridecadiene	44.98	2.405	—	—	—
97	2-Methyl-1-hexadecanol	45.3	—	—	—	0.33
98	cis-1-Chloro-9-octadecene	45.724	0.303	—	—	—
99	2-Tridecanone	45.901	0.67	—	0.77	0.252
100	Pentadecane	46.038	—	0.421	—	1.486
101	Tridecanal	46.519	1.079	—	—	—
102	[1R-(1α,3α,7α)]-1,2,3,6,7,7-hexahydro-2,2,4,7-tetramethyl-1,3-ethano-3H-indene	46.908	—	0.321	—	—
103	[1S-(1α,3β,4α,8β)]-Decahydro-1,5,5,8-tetramethyl-1,4-methanoazulen-3-ol	46.948	0.776	—	—	—
104	2-Hexadecanol	47.921	—	—	—	0.466
105	Tetradecanal	49.134	5.888	0.16	—	—
106	6-Trimethylsilyl-1H-indole-2,3-dione	49.345	—	—	0.599	0.359
107	Hexadecane	50.078	—	0.561	—	—
108	7-Methyl-Z-tetradecen-1-ol acetate	52.441	—	—	—	0.378
109	2-Methyl-1-Hexadecanol	52.967	—	—	—	0.782
110	Hexadecanal	53.059	10.889	—	—	—
111	Hexadecyl-oxirane	53.334	7.203	—	—	—
112	N-(Acetoxymethyl)-2-(2,4-pentadienyl)-cyclohexanecarboxamide	53.637	—	—	0.458	—
113	2-Pentadecanone	53.854	1.193	—	—	—
114	Nonadecane	53.871	—	0.247	—	—
115	3-Acetamido-2-methallylphenol	53.906	—	—	1.316	—
116	Pentadecanal	54.449	—	0.26	—	—
117	Naphthalene,decahydro-1,1,4a-trimethyl-6-methylene-5-(3-methylene-4-pentenyl)	54.684	—	—	—	0.254
118	Octadecanal	56.727	0.802	0.824	—	—
119	cis-9-Hexadecenal	57.648	—	0.41	—	—
120	Propanoic acid 2-(3-acetoxy-4,4,14-trimethylandrost-8-en-17-yl)	61.39	—	—	—	0.124
121	Tetradecyl-oxirane	61.396	—	0.042	—	—
122	n-Hexadecanoic acid	62.065	—	0.703	—	—
123	(Z)-9-Octadecenal	62.392	0.888	0.721	—	—
124	1,2-Dedihydro-perhydro-histrionicotoxin methyl ether	65.716	—	—	—	0.444
125	Heptacosane	66.443	—	—	—	0.112
126	Tricosane	66.454	—	0.093	—	—
127	3-Methyl-2-[(trimethylsilyl)oxy]-benzoic acid, trimethylsilyl ester	66.569	—	0.024	—	—
Total			37 types	44 types	47 types	53 types

Note: TDL, TDP, TDA (F), TDA (M), respectively, represent the larva powder, pupa powder, female adult powder, and male adult powder of *T. dichotomus*, and RT represents the retention time.

**Table 2:** Content of total flavonoids, total polyphenols, and total triterpenes of ethanol extract and total polysaccharides of water extract from TDL, TDP, TDA (M), and TDA (F).

Compound	Content (mg/g)			
	TDL	TDP	TDA (M)	TDA (F)
Flavonoids	155.7	13.9	35.9	34.5
Polyphenols	25.8	23.4	25.5	67.6
Triterpenes	22.8	39.5	15.0	18.0
Polysaccharides	33.5	23.0	23.3	18.9

**Table 3:** Composition and content of amino acids in TDA (M) and TDA (F) powders.

No.		Amino Acid	Content (mg/g)	
			TDA (M)	TDA (F)
440 nm	1	Asp	97.27	88.36
	2	Thr	32.72	28.07
	3	Ser	52.98	48.39
	4	Glu	136.71	117.11
	5	Gly	128.42	129.63
	6	Ala	116.92	119.15
	7	Cys	0.72	0.65
	8	Met	9.75	7.90
	9	Ile	53.08	48.90
	10	Leu	85.55	81.27
	11	Tyr	34.82	30.33
	12	Phe	20.83	16.79
	13	His	203.48	217.17
	14	Arg	40.39	34.70
570 nm	15	Pro	88.1	87.48

(Note: TDA (F) and TDA (M), respectively, represent the female adult powder and male adult powder of *T. dichotomus*).

**Table 4:** Content of inorganic elements.

Name	Content (mg/g) × 10^−6^											
	Mg	Zn	Ga	Al	Bi	Li	Be	B	Ti	V	Cr	Mn
TDL	135.43	3.61	1.19	25.15	5.41	0.08	-	-	14.62	0.19	0.61	13.62
TDP	113.23	2.14	1.26	-	11.14	-	-	0.16	1	-	-	4.18
TDA (M)	32.59	6.18	1.21	2.63	9.14	-	-	-	1.39	-	-	4.72
TDA (F)	132.54	5.33	1.39	-	25.96	0.02	-	-	0.92	-	-	5.1
	Co		Ni	Cu	As	Sr	Cd	Sn	Sb	Ba	TI	Pb
TDL	0.14		1.58	2.52	0.71	2.72	0.28	-	-	3.5	-	0.23
TDP	0.03		0.6	2.47	0.94	0.3	0.11	-	-	0.33	-	-
TDA (M)	-		0.82	3.32	1.82	0.2	-	-	-	0.07	-	-
TDA (F)	-		0.71	3.84	2.12	0.2	-	-	-	-	-	0.28

(Note: TDL, TDP, TDA (F), and TDA (M), respectively, represent the larva powder, pupa powder, female adult powder, and male adult powder of *T. dichotomus*).

**Table 5:** Anticancer activity of ethanolic extracts (data are means ± SD, n = 3).

Compound	Concentration	Cell Inhibition (%)			
		MKN-45	K-562	5637	239T
TDA (M, C)	0.5 mg/mL	86.22 ± 1.19	85.42 ± 0.63	88.15 ± 3.27	97.23 ± 0.60
TDA (F, C)	0.5 mg/mL	—	81.28 ± 1.19	—	—
Dox	10 μM	79.97 ± 1.95	78.13 ± 0.52	96.78 ± 0.37	95.84 ± 1.32

(Note: TDA (F, C) refers to the ethanol extracts of female adults of *T. dichotomus*; TDA (M, C) refers to the ethanol extracts of male adults of *T. dichotomus*).

**Table 6:** Free radical scavenging IC50 values for *T. dichotomus* (data are means ± SD, n = 3).

Free Radical	IC_50_ (mg/mL)								
	TDL (C)	TDL (W)	TDP (C)	TDP (W)	TDA (F, C)	TDA (F, W)	TDA (M, C)	TDA (M, W)	Vc
DPPH·	1.155 ± 0.188	2.090 ± 0.307	1.142 ± 0.653	2.315 ± 0.469	0.672 ± 0.306	1.220 ± 0.222	1.008 ± 0.502	1.677 ± 0.320	0.025 ± 0.163
·OH	-	-	-	-	-	1.696 ± 0.649	1.573 ± 0.375	-	0.016 ± 0.312
ABTS ^+^ ·	1.294 ± 0.114	1.062 ± 0.100	1.436 ± 0.290	2.34 ± 0.524	2.19 ± 0.701	0.589 ± 0.074	0.479 ± 0.047	0.736 ± 0.470	0.015 ± 0.113
·O_2_^−^	1.220 ± 0.492	-	1.416 ± 0.504	-	-	1.504 ± 0.309	0.773 ± 0.133	1.791 ± 0.552	0.031 ± 0.136

(Note: IC_50_ is the half-maximal inhibitory concentration. Vc is the positive control. *-* indicates not identified).

## Data Availability

All data included in this study are available upon request by contact with the corresponding author.
